# Thiolated Nanoparticles for Biomedical Applications: Mimicking the Workhorses of Our Body

**DOI:** 10.1002/advs.202102451

**Published:** 2021-11-12

**Authors:** Nathalie Hock, Giuseppe Francesco Racaniello, Sam Aspinall, Nunzio Denora, Vitaliy V. Khutoryanskiy, Andreas Bernkop‐Schnürch

**Affiliations:** ^1^ Thiomatrix Forschungs und Beratungs GmbH Trientlgasse 65 Innsbruck 6020 Austria; ^2^ Department of Pharmacy – Pharmaceutical Sciences University of Bari “Aldo Moro” Bari 70125 Italy; ^3^ Reading School of Pharmacy University of Reading Whiteknights PO Box 224, Room 122 (Chemistry and Pharmacy Building) Reading RG66DX UK; ^4^ Department of Pharmaceutical Technology, Institute of Pharmacy University of Innsbruck Innrain 80/82 Innsbruck 6020 Austria

**Keywords:** bioadhesion, biosensing, diagnostics, drug delivery, mucoadhesion, nanoparticles, quantum dots, thiolated polymers, thiolation, thiomers

## Abstract

Advances in nanotechnology have generated a broad range of nanoparticles (NPs) for numerous biomedical applications. Among the various properties of NPs are functionalities being related to thiol substructures. Numerous biological processes that are mediated by cysteine or cystine subunits of proteins representing the workhorses of the bodies can be transferred to NPs. This review focuses on the interface between thiol chemistry and NPs. Pros and cons of different techniques for thiolation of NPs are discussed. Furthermore, the various functionalities gained by thiolation are highlighted. These include overall bio‐ and mucoadhesive, cellular uptake enhancing, and permeation enhancing properties. Drugs being either covalently attached to thiolated NPs via disulfide bonds or being entrapped in thiolated polymeric NPs that are stabilized via inter‐ and intrachain crosslinking can be released at the diseased tissue or in target cells under reducing conditions. Moreover, drugs, targeting ligands, biological analytes, and enzymes bearing thiol substructures can be immobilized on noble metal NPs and quantum dots for therapeutic, theranostic, diagnostic, biosensing, and analytical reasons. Within this review a concise summary and analysis of the current knowledge, future directions, and potential clinical use of thiolated NPs are provided.

## Graphical abstract

Among the various properties of nanoparticles (NPs) that are used for biomedical applications are functionalities being related to thiol substructures. This review focuses on the interface between thiol chemistry and NPs and provides a concise summary of the current knowledge, future directions, and potential clinical use of thiolated NPs.

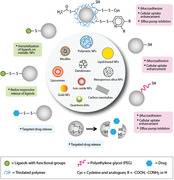

## 1 Introduction

**Figure 1:**
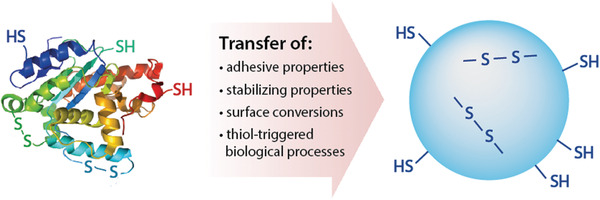
Numerous biological processes that are mediated by cysteine or cystine subunits of proteins representing the workhorses of our bodies can be transferred to NPs.

Advances in nanotechnology have generated a broad range of nanoparticles (NPs) for numerous biomedical applications. Smart NPs have the potential to protect drugs against the environment, to shuttle drugs to their target site, to obtain detailed cellular and molecular imaging of diseased tissues and to biosense biological analytes. Among the various characteristics of NPs such as size and shape, in particular certain chemical substructures and functional groups on nanostructured materials governing interactions with the biological environment are responsible for their performance. Poly(ethylene glycol) (PEG) substructures, for instance, provide stealth features avoiding opsonization and prolonging the systemic circulation of NPs unless repeatedly administered^[^ ^1^, ^2^ ^]^ folic acid substructures have shown potential for tumor targeting^[^ ^3^ ^]^ and cleavable phosphate groups can provide a charge conversion of NPs from negative to positive enabling them to overcome the polycation dilemma.^[^ ^4^, ^5^ ^]^ Another chemical substructure performing complex functions on NPs are thiols. Numerous biological processes that are mediated by cysteine or cystine subunits of proteins representing the workhorses of our bodies can be transferred to NPs as illustrated in **Figure** **1**.^[^ ^6^ ^]^ Mimicking such biological processes opens the door for an ever expanding field of biomedical applications.

The capability of thiols to form disulfides—perhaps the most important bridging structure in nature—with cysteine subunits of endogenous proteins render them unique among functional groups that are utilized in nanotechnology.

i) By the formation of disulfide bonds thiolated NPs can be tightly bound to biological surfaces that exhibit cysteine‐rich substructures such as the mucus gel layer,^[^ ^7^ ^]^ keratinized tissues and cell membranes.
ii) Furthermore, disulfide bonds can be formed within thiolated NPs such as within proteins. Drugs being encapsulated in the 3D network of NPs formed by a disulfide crosslinking can be released at hypoxic target sites due to a reductive cleavage of these bonds.
iii) Similar to proteins that can alter their surface properties due to the reduction of disulfide bonds triggering conformational changes, thiolated NPs can alter their surface properties under reducing conditions. NPs that are PEG‐coated via a disulfide linkage, for instance, alter their surface under reducing conditions due to the release of this hydrophilic coating.^[^ ^8^ ^]^
iv) Even thiol‐triggered biological processes can be transferred to NPs. By decorating NPs with thiols, for instance, an improved internalization by target cells via a thiol mediated trigger can be achieved.^[^ ^9^, ^10^ ^]^
v) Additionally, utilizing the high binding affinity of thiols to noble metals various types of thiol bearing compounds such as drugs, targeting ligands, biological analytes and enzymes can be immobilized on gold and silver NPs for therapeutic, theranostic, diagnostic, biosensing, and analytical reasons.

Within this review an overview about the different types of thiolated NPs and methods for their preparation as well as characterization is provided. The properties of these NPs gained by thiolation are highlighted providing insights in the chemistry behind. Moreover, an overview and outlook on the numerous biomedical applications of thiolated NPs is given.

## 2 Thiolation of Nanoparticles

Generally, NPs can be thiolated by the introduction of free thiol groups, whose reactivity depends on their pKa. The pKa of appropriate sulfhydryl ligands ranging from 4.3 to 10.5 has been reviewed in detail previously.^[^ ^11^ ^]^ Since these thiol groups are considerably unstable forming disulfide bonds under oxidizing conditions, they are in many cases S‐protected. As illustrated in **Table** **1** such an S‐protection is achieved by the formation of disulfide bonds with 2‐mercaptopyridine and analogues. Because of the electron withdrawing effect of *π* system of the pyridine substructure such disulfides are preactivated reacting rapidly with endogenous thiols. When a too rapid reaction of S‐protected thiols is disadvantageous, less reactive S‐protective groups such as cysteine and analogues exhibiting a lower or no electron withdrawing effect or thioacetates can be used.

**Table 1:** Different ligands that are used for the thiolation of NPs

Thiol ligand	Chemical structure	Frequently used ligands	Stable toward oxidation	Reactive toward	Reactivity
Free thiols	—SH	Cysteine; cysteamine; thioglycolic acid;	No	Disulfides	Depending on the pKa of thiol group and pH of medium
Disulfides	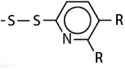 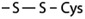	Highly reactive ligands: disulfides with mercaptonicotinic acid, mercaptonicotinaminde; Low reactive ligands: disulfides with cysteine and analogues;	Yes	Thiols	Depending on the electron withdrawing effect of the groups neighbouring the disulfide bond
Thioesters	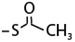	Thioacetate	Yes	Nucleophiles, even water	Depending on pH of medium

### 2.1 Thiolated Polymeric Nanoparticles

For the preparation of thiolated polymeric NPs thiolated polymers can be used as starting material and formulated to NPs or preformed polymeric NPs can be coated with thiolated polymers. The preparation of NPs based on already preformed thiolated polymers that can be synthesized via numerous techniques as having been reviewed previously^[^ ^11^, ^12^ ^]^ is generally preferred over other techniques. As various thiolated polymers such as thiolated chitosan,^[^ ^13^ ^]^ thiolated hyaluronic acid,^[^ ^14^ ^]^ and thiolated polyacrylic acid^[^ ^15^ ^]^ were already subject of clinical trials and the manufacturing process for GMP material is established, the preparation of thiolated NPs based on such polymers is from the industrial point of view most straight forward. Preparation methods of NPs starting with preformed thiolated polymers include different nanoprecipitation, spray drying, self‐assembly, and emulsification techniques. When oxidizing conditions are provided during the preparation process, NPs are stabilized via the formation of intra‐ and interchain disulfide bonds.

#### 2.1.1 Nanoprecipitation

**Figure 2:**
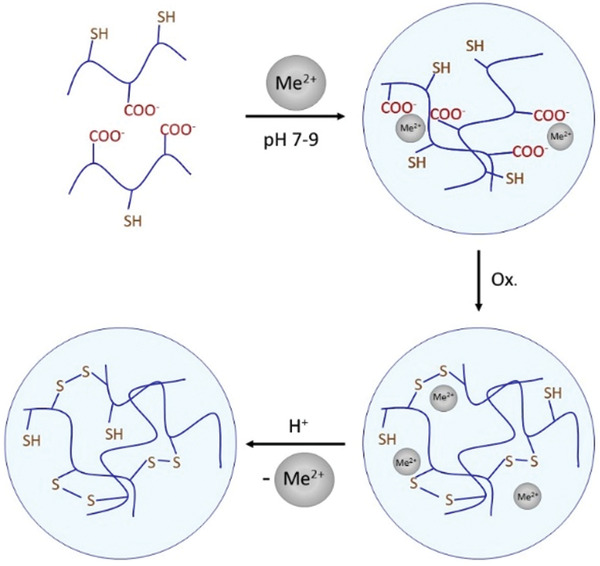
Formation of thiolated NPs via nanoprecipitation. First, thiolated polyacrylic acid is ionically crosslinked with a divalent cation (Me^2+^). The formed NPs are stabilized by disulfide bond formation under oxidizing conditions and the divalent cations are removed.^[^ ^18^ ^]^

Nanoprecipitation is mainly obtained by the addition of di‐ or trivalent cations to anionic thiolated polymers resulting in strong ionic interactions and the formation of NPs by coacervation. Thiolated poly(acrylic acid) NPs in a size range of 158–214 nm were, for instance, prepared by a simple coacervation with calcium chloride.^[^ ^16^ ^]^ Using di‐ or trivalent metal ions such as Ca^2+^, Mg^2+^, Zn^2+^, Fe^3+^ or Al^3+^ ions for this process offers the additional advantage that they can be removed via complexation with auxiliary agents like EDTA after the preparation process. In this way drawbacks of counterions such as inhibited mucoadhesive properties can be avoided.^[^ ^17^ ^]^ As illustrated in **Figure** **2**, thiolated polyacrylate NPs were prepared via nanoprecipitation of thiolated polyacrylic acid with calcium ions causing an ionic crosslinking of the polymer and the formation of particles in the size range of 200–300 nm. In the following step these NPs were stabilized via the formation of inter‐ and intrachain disulfide bonds in the presence of hydrogen peroxide. Finally, calcium ions were removed.^[^ ^18^ ^]^

In case of cationic thiolated polymers, nanoprecipitation can be achieved by the addition of di‐ or trivalent anions such as sulfate or tripolyphosphate.^[^ ^19^, ^20^ ^]^ Thiolated chitosan NPs containing insulin were formed due to the addition of tripolyphosphate.^[^ ^21^ ^]^ Apart from di‐ or trivalent counterions, nanoprecipitation can also be achieved with oppositely charged polymers. For example, NPs were formed by co‐precipitating thiolated poly(acrylic acid) with the cationic polymer chitosan^[^ ^22^ ^]^ and thiolated chitosan with poly(malic acid)^[^ ^23^ ^]^ or with sodium alginate.^[^ ^24^ ^]^ Using oppositely charged polymers for an ionic crosslinking, however, can result in a comparatively big particle size. In order to avoid this, polymers that are used for coacervation can be depolymerized. Zambito et al. synthesized thiolated quaternary ammonium–chitosan conjugates from depolymerized chitosan and studied the effect of reaction conditions, especially temperature, on the formation of NPs.^[^ ^25^ ^]^ Apart from polymer chain length and temperature, various further factors such as charge and weight ratio of polymers, pH of the solution, ionic strength as well as the preparation technique affect particle size.^[^ ^26^ ^]^ Furthermore, therapeutic agents per se can be used for nanoprecipitation. Because of their polyanionic character in particular DNA‐ and RNA‐based drugs can be coacervated with cationic thiolated polymers. Thiolated chitosan, for example, was coacervated with pDNA leading to NPs with an average size of 125 nm.^[^ ^27^ ^]^

Nanoprecipitation can also be combined with spray drying techniques. The generation of droplets through a piezoelectric driven actuator that operates at a specific ultrasonic frequency results in an ultrafine particle size. The dried particles are electrostatically charged and collected on the surface of the collection electrode with minimal particle waste and high formulation yields.^[^ ^28^ ^]^ However, particle sizes are often high being in the range of 500–1000 nm^[^ ^29^ ^]^ and depending on the thiolated starting material they can even exceed particle sizes of 1 *µ*m.^[^ ^30^ ^]^

#### 2.1.2 Self‐Assembly

**Figure 3:**
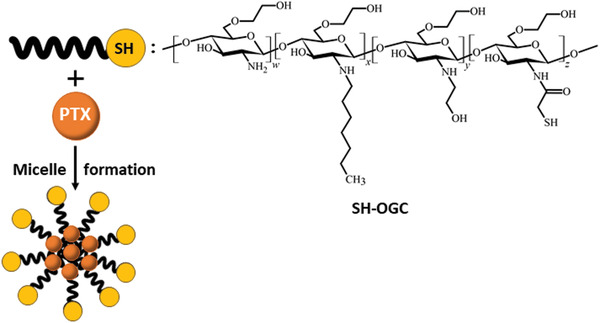
Micelle formation of thiolated N‐octyl‐O,N′‐glycol chitosan (SH‐OGC) and incorporation of the hydrophobic drug paclitaxel (PTX) during this self‐assembly process in aqueous media. Reproduced with permission.^[^ ^31^ ^]^ Copyright 2018, Elsevier.

Amphiphilic thiolated polymers were shown to self‐assemble to form micelles. In aqueous media the hydrophobic substructures of these polymers interact forming hydrophobic cores being surrounded by their hydrophilic substructures bearing thiol groups. As illustrated in **Figure** **3** thiolated N‐octyl‐O, N′‐glycol chitosan readily formed micelles in aqueous media solubilizing the hydrophobic drug paclitaxel in the formed hydrophobic inner core, whereas thiol groups are presented on the hydrophilic surface.^[^ ^31^ ^]^ In other studies hydrophobic substructures were introduced in thiolated chitosan by the covalent attachment of stearic acid and lauric acid being responsible for the self‐assembly of these polymers to micelles.^[^ ^32^, ^33^ ^]^ Other polysaccharides such as alginate were also shown to assemble to NPs by the introduction of hydrophobic groups. Chang et al. formed thiolated alginate NPs applying just ultrasound to an aqueous solution of the thiolated polymer that enhanced the self‐assembly process.^[^ ^34^ ^]^ As thiol groups were attached to the polymeric backbone via a hydrophobic thiol‐bearing ligand, namely, 4‐aminothiophenol, hydrophobic substructures were introduced forming the hydrophobic core of NPs in aqueous media. Similarly, Qiu et al. synthesized mPEG‐P(Phe‐*co*‐Cys) copolymers by ring‐opening polymerization of L‐Phe N‐carboxy anhydride and L‐Cys N‐carboxy anhydride. The copolymer self‐assembled into spherical NPs in aqueous solution with a diameter of 48 nm forming stabilizing disulfide bonds within its hydrophobic core.^[^ ^35^ ^]^ On contrary, in case of lipoic acid PEG‐*b*‐poly(d,l‐lactide) micelles sulfhydryl groups were orientated to the aqueous phase and the hydrophobic core was formed by the poly(d,l‐lactide) substructure.^[^ ^36^ ^]^ All these amphiphilic thiolated polymers are based on a comparatively complex chemistry for which the scale‐up and registration process is time consuming and costly. Therefore, the probability for self‐assembled thiolated NPs to enter clinical trials is comparatively low.

#### 2.1.3 Nanoparticle Coating with Thiolated Polymers

**Figure 4:**
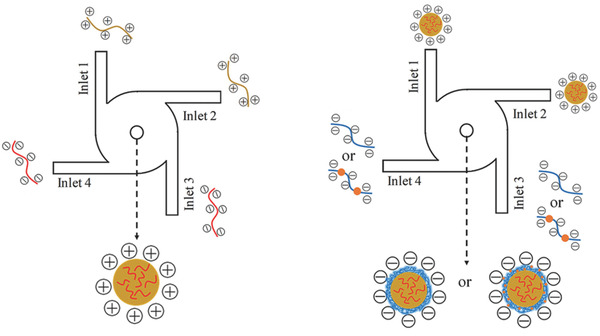
Schematic representation of the two‐step FNC platform for formation of thiolated polymer coated NPs. In the first step, positively charged NPs are prepared by mixing negative charged with positive charged solutions under a turbulent mixing condition using an FNC apparatus. In the second step, these NPs are coated with a polyanionic thiolated polymer to form thiolated NPs. Reproduced with permission.^[^ ^39^ ^]^ Copyright 2018, Wiley‐VCH GmbH.

Several drug‐loaded thiolated nanoparticles were generated using a two‐step termed flash nanocomplexation (FNC) process as illustrated in **Figure** **4** in a miniature mixing chamber that can achieve rapid and homogeneous mixing of two or more oppositely charged aqueous solutions of polyelectrolytes.^[^ ^37^, ^38^ ^]^ Tian et al. synthesized core–shell nanoparticles with thiolated hyaluronic acid (HA‐SH) used as a coating via a flash nanocomplexation process to improve oral insulin delivery efficiency.^[^ ^39^ ^]^ A positively charged NP core was generated first by coacervation of insulin and chitosan modified with N‐(2‐hydroxypropyl)‐3‐trimethyl ammonium chloride (HTCC), followed by surface coating with HA‐SH. The positively charged HTCC solution and negatively charged insulin solution were introduced through inputs 1, 2 and inputs 3, 4, respectively. The average size of the obtained NPs was 75 nm. In the second step, these NPs were coated with polyanionic HA‐SH by surface charge neutralization. The NP dispersion (inlets 1 and 2) and HA‐SH solution (inlets 3 and 4) were separately introduced into the FNC chamber to generate HA‐SH coated NPs exhibiting a spherical shape with a core–shell structure.

Another approach is based on a simultaneous NP formation and coating process. The formation of NPs is provided by a radical emulsion polymerization of alkylcyanoacrylates to produce core–shell NPs coated with thiolated polysaccharides. The process is initiated by the thiolated polysaccharide on which radicals are created by reaction with cerium(IV) ions in an aqueous acidic medium. These radicals cause the polymerization of monomers forming linear block copolymers. Since the reaction occurs in aqueous media, the hydrophobic polymer tends to go inside the NPs at the same time as the particles are formed creating a hydrophobic core coated with the hydrophilic thiolated polysaccharide.^[^ ^40^, ^41^, ^42^ ^]^ Akhlaghi et al., for instance, prepared poly(methyl methacrylate) NPs loaded with paclitaxel that were coated with thiolated chitosan by this radical polymerization method.^[^ ^43^ ^]^

#### 2.1.4 Thiolation of Dendrimers

A special type of thiolated polymeric NPs are thiolated dendrimers. Generally, dendrimers are highly branched natural and synthetic macromolecules forming spherical particles that have been widely evaluated as delivery systems for a variety of therapeutic and diagnostic applications.^[^ ^44^ ^]^ Due to their spacious architecture, drugs can be incorporated into these comparatively small nanocarrier systems. The unique structural features of dendrimeric and hyperbranched macromolecules, which have a number of chains ends and a high degree of branching, leads to different physical properties compared to conventional linear polymers. In particular, they have been studied as nanocarriers for chemotherapeutic agents or bioactive materials and as solubilizing agents.^[^ ^45^ ^]^ In particular, when all end groups of dendrimers are thiolated, these carrier systems exhibit a comparatively high thiol density on their surface. Day et al. covalently attached S‐protected thiol groups—thioacetate groups—to generation 3 polyamidomaine (PAMAM) dendrimers.^[^ ^46^ ^]^ In another study cysteamine was covalently attached to generation 3.5 PAMAM dendrimers.^[^ ^45^ ^]^ Thiol ended carbosilane dendrimers were synthesized by addition of the thiol functionality to the C═C bond of carbosilane dendrimers with terminal allyl functions via a free‐radical‐mechanism.^[^ ^47^ ^]^ Thiol ended carbosilane dendrimers were also synthesized by nucleophilic substitution of (chloromethyl)silyl‐terminated dendrimers with thioacetate followed by deprotection of thiol groups upon treatment with hydrogen chloride providing a multivalent platform for the binding of various ligands of biological interest via thiol–ene reactions.^[^ ^48^ ^]^ Furthermore, natural dendrimers were conjugated with high thiol content due to their polyvalent nature. Glycogen–cysteamine conjugates were synthesized through a first step of oxidative ring opening by applying increasing concentrations of sodium periodate, to obtain polymers with different degrees of oxidation, and a second step of reductive amination with a constant amount of cysteamine. The introduction of thiol groups on the polymer changed the characteristics of the polysaccharide by improving mucoadhesion properties.^[^ ^49^ ^]^

### 2.2 Thiolation of Lipid‐Based Nanoparticles

Most lipid‐based NPs are thiolated via surfactants that bear thiol groups on their hydrophilic head group. When such thiolated surfactants as listed in **Table** **2** are added to lipophilic formulations forming NPs in aqueous media, thiol groups assemble on the surface of these particles. An advantage of lipid‐based thiolated NPs lies in the great flexibility for the design of such systems, as numerous surfactants and lipophilic excipients can be combined on purpose resulting in NPs of very different properties. Independently from the applied preparation method such as melt‐emulsification method for solid lipid nanoparticles and nanostructured lipid carriers (NLC)^[^ ^50^ ^]^ or thin‐film hydration and extrusion method for unilamellar liposomes^[^ ^51^ ^]^ this process works for all types of lipid based NPs in the same way. Consequently, there are no different or additional preparation techniques needed. Shen et al. designed a cysteine–polyethylene glycol stearate conjugate as illustrated in Table 2 and prepared NLC with this thiolated excipient by a fusion‐emulsification method.^[^ ^50^ ^]^

**Table 2:** Lipophilic thiolated compounds used for the formation of lipid‐based thiolated nanoparticles

Thiolated surfactant	Structure	Lipid‐based nanoparticles	Reference
(R)‐2‐Amine‐3‐mercaptopropanoate of octadecyl 2,3‐dihydroxypropanoate	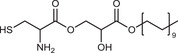	Solid lipid nanoparticles	^[^ ^57^ ^]^
S‐Protected thiolated palmitic acid	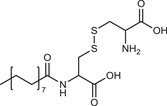	Nanostructured lipid carriers	^[^ ^52^ ^]^
Cysteine‐polyethylene glycol stearate	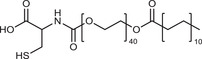	Nanostructured lipid carriers	^[^ ^50^, ^51^, ^52^, ^53^, ^54^, ^55^, ^56^, ^57^, ^58^, ^59^ ^]^
N‐Acetyl‐l‐cysteine‐polyethylene glycol stearate	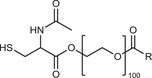	Nanostructured lipid carriers	^[^ ^60^ ^]^
Thioglycolic acid–octylamine conjugate	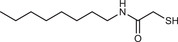	SEDDS	^[^ ^61^ ^]^
Cysteine‐functionalized phospholipid (Cys‐PEG‐DSPE)	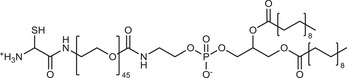	Liposomes	^[^ ^62^ ^]^
Cholesterol (Chol)‐integrated tetraether lipid comprising of a cleavable disulfide bond	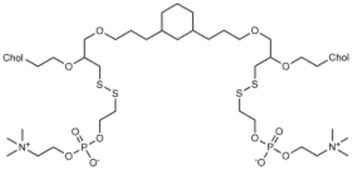	Liposomes	^[^ ^63^ ^]^
1,2‐Dipalmitoyl‐sn‐glycero‐3‐phosphothioethanol	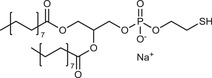	Liposomes	^[^ ^51^ ^]^
Multifunctional thiolated amino lipid	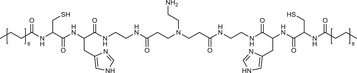	Lipid‐based siRNA delivery systems	^[^ ^64^ ^]^
Lipoic acid derivative	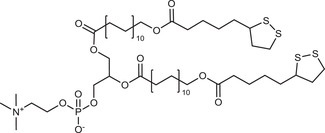	Liposomes	^[^ ^54^ ^]^
Lipoic acid derivative	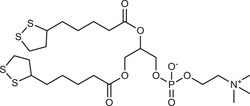	Liposomes	^[^ ^53^ ^]^
Lipoic acid derivatives	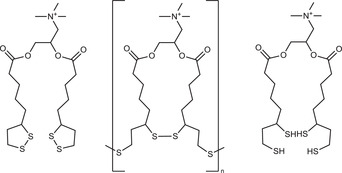	Lipophilic complexes with DNA	^[^ ^55^ ^]^

As thiol groups might be oxidized during the preparation process, however, either a pH < 5 is needed in order to keep the formation of thiolate anions representing the reactive species of thiols for oxidation at a minimum or the preparation process is performed under inert conditions. Alternatively, S‐protected thiolated surfactants such as S‐protected thiolated palmitic acid can be used.^[^ ^52^ ^]^ If thiol groups are intended to remain in the lipophilic phase in order to stabilize the formulation via a disulfide crosslinking^[^ ^53^, ^54^ ^]^ or to provide a redox‐triggered drug release^[^ ^55^ ^]^ they can also be attached to the lipophilic tail region of surfactants as shown for various lipoic acid derivatives (Table 2). In case of drugs that are released from lipid‐based NPs under reducing conditions, lipophilic excipients to which drugs are bound via a disulfide linkage can be simply added to the lipophilic phase during the preparation process.^[^ ^56^ ^]^

Furthermore, lipid‐based NPs can be coated with thiolated polymers as listed in **Table** **3**. Such a coating can be achieved by making use of ionic interactions between the surface of lipid‐based NPs and the coating material. Li et al., for instance, designed curcumin loaded liposomes with a zeta potential of −44 mV. Due to ionic interactions between this anionic surface charge and cationic chitosan–cysteine conjugate, these liposomes were efficiently coated with this thiolated polymer causing a shift in zeta potential to +37 mV. In order to further stabilize this coating via a disulfide crosslinking thiol groups were oxidized after the coating process.^[^ ^65^ ^]^ Liu et al. developed NLCs using a fusion‐emulsification technique labeled with coumarin‐6 (C6), coated with a chitosan–N‐acetyl cysteine conjugate for ocular delivery.^[^ ^66^ ^]^

**Table 3:** Lipophilic NPs coated with a thiolated polymer

Lipid‐based nanoparticles	Coating material	Type of surface coating	Function	Reference
Liposomes	Thiolated polyacrylic acid	Ionic coating	Mucoadhesive	^[^ ^67^ ^]^
	Chitosan–cysteine	Ionic coating	Improved physical stability	^[^ ^72^ ^]^
	Chitosan–cysteine	Ionic coating	Improved physical stability	^[^ ^65^ ^]^
	Chitosan–glutathione	Ionic coating	Protection towards autoxidation of the incorporated drug	^[^ ^73^ ^]^
	Chitosan–thioglycolic acid–Pluronic F127	Ionic coating	Mucoadhesive	^[^ ^74^ ^]^
	S‐Protected chitosan–thioglycolic acid	Ionic coating	Permeation enhancing	^[^ ^75^ ^]^
Nanostructured lipid carriers	Chitosan–N‐acetyl cysteine	Ionic coating	Mucoadhesive Permeation enhancing	^[^ ^66^, ^76^, ^77^ ^]^
Solid lipid NPs	(S‐Protected) chitosan–cysteine	Ionic coating	Mucoadhesive	^[^ ^78^ ^]^
Self‐emulsifying drug delivery systems (SEDDS)	S‐Protected thiolated Eudragit L100‐55	Ionic coating	Mucoadhesive	^[^ ^70^ ^]^
	Chitosan–N‐acetyl cysteine‐6‐mercaptonicotinamide	Ionic coating	Mucoadhesive	^[^ ^69^ ^]^
	S‐Protected thiolated acrylate/C10‐30 alkyl acrylate crosspolymer	Coating via lipophilic anchors	Mucoadhesive	^[^ ^71^ ^]^

In most studies lipid‐based NPs exhibiting a negative zeta potential are coated with cationic thiolated polymers and in particular with thiolated chitosans. In a few studies, however, it was also shown that lipid‐based NPs exhibiting a positive zeta potential can be coated with anionic thiolated polymers. Cationic sub‐micrometer‐sized liposomes, for instance, were successfully coated with thiolated polyacrylic acid.^[^ ^67^ ^]^ As these coated liposomes show an anionic zeta potential they are comparatively more bioinert than others keeping in mind that most surfaces in the bioenvironment are anionic.^[^ ^5^ ^]^ Bioinert properties allow NPs to move more freely across mucus and the extracellular matrix. As the cellular membrane has also an anionic character, however, cellular uptake of anionic NPs is generally lower than that of cationic ones.^[^ ^68^ ^]^ Furthermore, hydrophobic ionic complexes of thiolated polymers with lipophilic counter ions can be formed that are assembling on the interface between the lipid‐based NPs and the aqueous medium. Thiolated chitosan, for instance, was complexed with sodium dodecyl sulfate. The resulting hydrophobic complex was used as coating material for self‐emulsifying drug delivery systems (SEDDS).^[^ ^69^ ^]^ Similarly, a thiolated polymethacrylate was complexed with benzalkonium chloride and used as coating material for SEDDS.^[^ ^70^ ^]^

Alternatively lipophilic anchoring groups such as fatty acid can be covalently attached to polymers. A thiolated acrylate crosspolymer with C10‐30 alkyl side chains, for instance, was used to coat SEDDS.^[^ ^71^ ^]^

### 2.3 Thiolation of Carbon Nanotubes

**Figure 5:**
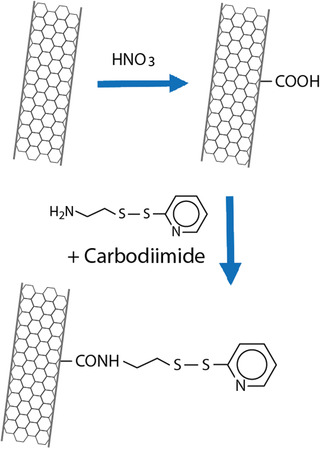
Synthetic pathway for the thiolation of carbon nanotubes.^[^ ^80^ ^]^

As most of the carbon nanotubes' grafting reactions require harsh conditions, it is difficult to attach thiol groups onto carbon nanotubes. The likely most promising and effective approach to address this issue is the introduction of other functional groups such as hydroxyl or carboxyl groups under harsh conditions and to thiolate these functional groups in a second step under relatively mild conditions. The thiolation of carbon nanotubes starts therefore in case of most methods by an oxidation with of single‐ or multiwalled carbon nanotubes in concentrated nitric acid and hydrogen peroxide.^[^ ^79^, ^80^, ^81^ ^]^ This process results in the formation of hydroxyl and carboxyl groups that can be subsequently thiolated. Either hydroxyl groups are converted into thiols with P_4_S_10_ or thiourea^[^ ^79^ ^]^ or sulfhydryl ligands such as S‐(2‐aminoethylthio)‐2‐thiopyridine or 4‐amino‐thiophenol are covalently attached to the carboxyl groups via amide bond formation.^[^ ^80^ ^]^ In **Figure** **5** a representative synthetic pathway for the thiolation of carbon nanotubes is shown.

Alternatively, thiolated surfactants such as thiolated phospholipids can be noncovalently attached to carbon nanotubes making us of van der Waals and hydrophobic interactions between alkyl chains of these surfactants and the hydrophobic side wall of the nanotubes.^[^ ^82^ ^]^

### 2.4 Thiolation of Inorganic Nanoparticles

#### 2.4.1 Thiolated Silica and Titanium Dioxide Nanoparticles

For the preparation of inorganic NPs, various strategies have been developed for the grafting of thiol polymers or direct introduction of small thiol‐bearing moieties onto the particles surface. Inorganic NPs surfaces can be thiolated with a wide range of ligands, with layer‐by‐layer assembly protocols and bioconjugation being widely accepted techniques in nanoscience.^[^ ^83^, ^84^, ^85^, ^86^ ^]^ Silicas high surface area, silanol surface concentration, and highly interconnected open spaces in their 3D structure make it an ideal surface to be thiolated. Due to its low toxicity, good biocompatibility and their ability to be loaded up a variety of organic and inorganic compounds allowing for transport and controlled release, silica particles have many biomedical uses with thiolation being able to enhance these properties.^[^ ^87^ ^]^ Silica NPs have traditionally been prepared using the Stober method where tetraethyl orthosilicate is first hydrolyzed and then undergoes a condensation reaction in a protic solvent with ammonia as a catalyst.^[^ ^88^ ^]^ Moller et al. described the making of thiol‐functionalized 50 and 200 nm NPs by cocondensation of (3‐mercaptopropyl)trimethoxysilane (MPTS) with tetraethyl orthosilicate in the presence of surfactants and triethanolamine as the basic medium.^[^ ^89^ ^]^ From there nucleation and particle growth generate silica NPs. Nakamura and Ishimura demonstrated that thiol‐organosilica NPs can be made from MPTS, (3‐mercaptopropyl)trimethoxysilane, and (3‐mercaptopropyl)methyldimethoxysilane.^[^ ^90^ ^]^

**Figure 6:**
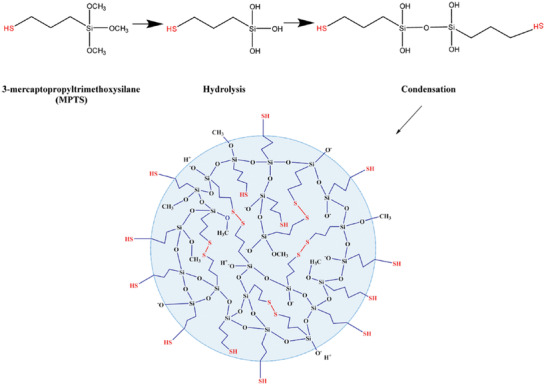
Formation of thiolated organosilica NPs from MPTS. Reproduced with permission.^[^ ^93^ ^]^ Copyright 2018, American Chemical Society.

Khutoryanskiy and co‐workers reported controlling the size of thiolated organosilica NPs from MPTS. They evaluated the effect of synthetic conditions, such as catalyst, solvent, temperature, and atmospheric oxygen on the characteristics of resulting NPs. The smallest nonporous particles (less than 50 nm) were obtained when NaOH was used as the catalyst and the self‐condensation of MPTS was conducted in dimethyl sulfoxide (DMSO) with air bubbling.^[^ ^91^, ^92^, ^93^ ^]^ Air bubbling was facilitating the formation of disulfide bonds between MPTS monomer molecules as illustrated in **Figure** **6**. Furthermore, they functionalized the surface of developed NPs with PEG or poly(2‐alkyl‐2‐oxazolines) and demonstrated their mucus‐penetrating properties^[^ ^94^, ^95^ ^]^

In another study mesoporous silica nanoparticles were also thiolated with MPTS. Via thiol–ene click chemistry a silyl ether prodrug of camptothecin being susceptible to acid‐catalyzed hydrolysis was covalently attached to these nanoparticles providing a controlled drug release under acidic conditions.^[^ ^96^ ^]^

Much like silica, titanium dioxide NPs can be readily functionalized with thiol‐bearing moieties. By grafting (3‐mercaptopropyl)trimethoxy silane to the surface, the thiolated TiNPs can be developed. These thiol groups act as a site for further reactions with other compounds undergoing thiol–ene Michael addition reactions.^[^ ^97^ ^]^ Work by Tedja et al. demonstrated that by thiolating TiO_2_ NPs with MPTS, before grafting the oligo(ethylene glycol) methyl ether methacrylate monomer onto the surface, a higher success rate was achieved compared with nonthiolated TiO_2_ NPs.^[^ ^97^ ^]^

#### 2.4.2 Thiolated Magnetic Nanoparticles

For magnetic NPs (MNPs), the low reactivity of the particles requires a different approach to functionalizing the surface with thiol groups. The MNPs usually consist of a metal, alloy or metal oxide core based on iron, nickel, manganese or cobalt core.^[^ ^98^ ^]^ Of these NPs, iron‐based MNPs and in particular superparamagnetic iron oxide (SPIO) NPs have been studied the most due to the toxic nature of cobalt and nickel and iron‘s increased biocompatibility over other magnetic metal NPs.^[^ ^99^ ^]^ To functionalize the MNPs, the surface must be coated in a shell that will allow the attachment of polymer chains to it. One approach is to create a silica shell around the MNPs using tetraethyl orthosilicate or sodium silicate in a sol–gel reaction allowing the coupling of silane agents to attach to the MNPs.^[^ ^100^, ^101^ ^]^ The silica coating allows the addition of silane coupling agent such as (3‐mercaptopropyl)triethoxysilane and 3‐aminopropyltriethoxysilane through a hydrolysis and condensation reaction onto the silica surface of the MNPs where they can be further functionalized to add thiol end groups.^[^ ^102^, ^103^, ^104^ ^]^ Activation of the silica shell maybe needed through washing with HCl solution as described by Zhang et al.^[^ ^102^ ^]^ Alternatively, a faster approach that eliminates a step is to graft a silane coupling agent that already has a thiol end group attached such as mercaptopropyl trimethoxysilane.^[^ ^103^, ^105^ ^]^

#### 2.4.3 Thiolated Noble Metal Nanoparticles

In particular research into gold NPs (AuNPs) and silver NPs (AgNPs) has seen substantial growth due to its application in electronics, nanotechnology, catalyst, and medicine.^[^ ^106^, ^107^ ^]^ Their size dependent properties, large surface to volume ratio and ease of functionalization make these NPs very desirable. Gold NPs are readily bound and capped to sulfur groups as the strong ionic and covalent characters of the Au—S bond help creating very stable NPs.^[^ ^108^ ^]^ The high affinity of gold for sulfur atoms means forming nanoparticles with free thiol groups on the surface requires a creative approach to overcome this. Due to the dynamic nature of the Au—S bond ligands on the surface of the AuNPs can be exchanged, which allow thiol end group ligands to be attached to the NPs.^[^ ^109^ ^]^ Ligands containing thiol groups are chemically bonded to the metal surface by coordination of the sulfur atom with epitaxial (hollow) surface sites.^[^ ^110^ ^]^ Initially, AuNPs must be functionalized with surface groups that can be exchanged before introducing thiol end group polymers. If a dithiol containing ligand is reacted with AuNPs, both thiol groups would bind to the surface potentially creating a ring structure. Binding precursor ligands to the surface of AuNPs overcomes this issue and allows further thiol surface functionalization. Pakiari and Jamshidi used a modified Brust method to synthesize thiolated gold NPs which does not require an ionic stabilizing/phase transfer agent, due to the synthesis being performed in water.^[^ ^111^ ^]^ They used tetrachloroauric acid (HAuCl_4_) and PEG which are both soluble in water allowing the avoidance of cationic surfactants which are toxic.^[^ ^111^ ^]^ Once the gold had been PEGylated, PEG double thiol was exchanged with PEG 2000 creating a thiolated surface.^[^ ^111^ ^]^ A wide range of thiol compounds used to prepare gold surfaces for biomodification but only a few have free thiol groups on the surface of the AuNPs. Dithiols bearing compounds are attached to gold particles in a way so as only one of the two thiol groups present is bound to the AuNPs, leaving a free SH group on the surface. This free thiol can attach NPs or metallic ions to self‐assembled monolayers.^[^ ^112^ ^]^ The addition of a dithiol such as 1,6‐hexanedithiol into an ethanolic solution containing an Au electrode under a nitrogen atmosphere will create a thiolated gold surface.^[^ ^113^ ^]^

AgNPs react with thiols in an almost identical way as AuNPs. Much like AuNPs, thiol groups help stabilizing silver NPs. Thiol groups can be easily grafted onto the surface of AgNPs using a modified two step Brust–Schriffin procedure.^[^ ^114^, ^115^ ^]^ A toluene solution containing allylmercaptane (AM) and AgNO_3_ was mixed before tetraoctylammonium bromide and NaBH_4_ in toluene was added.^[^ ^114^ ^]^ It was also found that an increase in Ag/AM ratio led to an increase in the thickness of the Ag_2_S layer, thus larger AgNPs were formed while the external AM layer remained unchanged.^[^ ^114^, ^116^ ^]^ A similar synthesis of thiolated AgNPs as demonstrated by Jeong et al. involves the dropwise addition of AgNO_3_ into a mixture of nonanethiol, ethanol, and NaBH_4_ whilst under vigorous stirring for 2 h before refrigerating at −18 °C for 4 h and washing to remove any free thiol or by‐products.^[^ ^117^ ^]^

#### 2.4.4 Thiolated Quantum Dots

Quantum dots (QDs) are semiconductor particles measuring a few nanometres in diameter. The quantum nature of these NPs gives them unique electronic and optical properties that are not exhibited by bulk semiconductors. Two types of QDs containing thiol groups will be looked at, those that are capped and stabilized by thiol groups and those that contain free thiols on the surface. Thiols that are used as capping molecules for QDs allow the synthesis of water‐soluble QDs due to the polar headgroup.^[^ ^118^ ^]^ Wuister et al. demonstrated that the use of thiols to cap CdTe QDs has a beneficial effect on the quantum efficiency (QE).^[^ ^118^ ^]^ In addition to this, high QE thiol‐capped CdTe QDs have also been reported by Weller and co‐workers.^[^ ^119^ ^]^ In another thiolated‐graphene quantum dots were prepared by hydrothermal pyrolysis of carbon source (citric acid) in the presence of reduced glutathione.^[^ ^120^ ^]^

**Figure 7:**
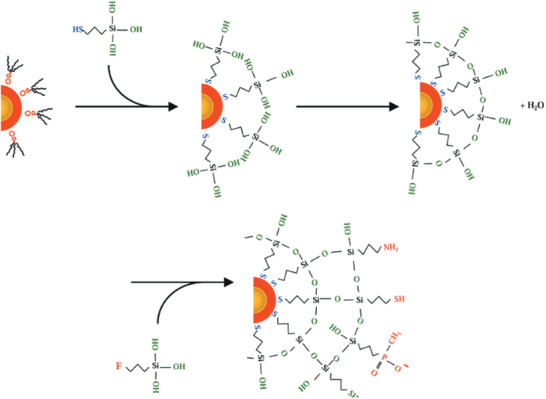
Deposition of a silica shell onto quantum dots and functionalization with mercaptopropyltris(methyloxy)silane. Reproduced with permission.^[^ ^122^ ^]^ Copyright 2001, American Chemical Society.

Zinc sulfide QDs can be produced with a thiolated surface by the formation of disulfide bonds with sulfur bearing polymer groups. A procedure used by Sharma et al. made ZnS uncapped QDs by passing nitrogen gas through a neutral solution of 0.1 m Zn(NO_3_)_2_ before adding an equal amount of 0.1 m Na_2_S dropwise and collecting the white precipitate of ZnS QDs after stirring for 1 h.^[^ ^121^ ^]^ From there, sulfur bearing groups like cysteine, mercaptopropyltris(methyloxy)silane and glutathione for example can be attached to the ZnS particles. Reacting QDs that have sulfur groups with dithiol groups cause both thiols to bind to the surface. To create a thiolated surface on QDs, they must first be encased in another shell. QDs with a ZnS shell can be encased again in a silica shell using mercaptopropyltris(methyloxy)silane as illustrated in **Figure** **7**, resulting in a reactive surface that can be functionalized the same way as silica NPs.^[^ ^122^ ^]^ Once fully coated with mercaptopropyltris(methyloxy)silane, further functionalization to form a thiolated surface can occur.

#### 2.4.5 Thiolated Nanodiamond Particles

Nanodiamonds (NDs) are carbon NPs that have a size smaller than 1 µm in diameter with a truncated octahedral architecture.^[^ ^123^ ^]^ Due to their biocompatibility, NDs can be used to deliver proteins, nucleic acids, and small molecules to patients and have seen a surge in research over the last few decades.^[^ ^124^ ^]^ Numerous techniques exist for surface modification and functionalization of NDs due to their ability to readily form covalent and noncovalent attachments with various polymers and biomolecules with functional groups able to attach.^[^ ^125^ ^]^

Three methods for the incorporation of thiol groups onto the surface of nanodiamonds will be discussed. The first method as described by Lud et al. involves the X‐ray‐ and electron‐induced modification of surface sulfonic monophenyl monolayers that have previously been covalently grafted onto the NDs surface to thiol groups.^[^ ^126^ ^]^ The second method first reported by Nakamura et al. is through the photolysis of elemental sulfur in carbon disulfide with diamond powder whilst being irradiated with a low pressure mercury lamp under an argon atmosphere.^[^ ^127^ ^]^ The third method as described by Tkachenko et al. used thiourea to replace tertiary or bridgehead hydroxyl groups thiols.^[^ ^128^ ^]^ The diamondoid and thiourea are refluxed in a mixture of glacial acetic acid and aqueous HBr under an argon atmosphere.^[^ ^128^ ^]^ The mixture is poured into cold NaOH solution and the suspension is acidified with H_2_SO_4_ before being extracted with chloroform to produce nanodiamond thiols.^[^ ^128^ ^]^ This method can also be applied to detonation nanodiamonds.^[^ ^129^ ^]^

## 3 Properties of Thiolated Nanoparticles

**Figure 8:**
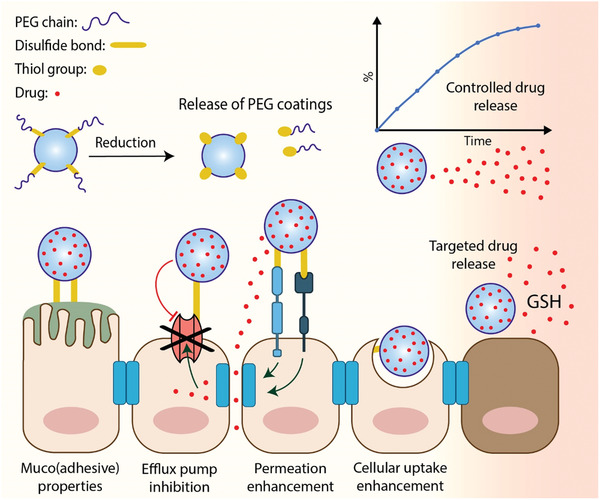
Overview about key properties of thiolated NPs for drug delivery.

Due to the immobilization of thiol groups on NPs, various properties such as adhesive properties, redox triggered functionalities, cellular uptake enhancing properties, and efflux pump inhibitory properties can be introduced or improved. An overview about key properties of thiolated NPs is provided in **Figure** **8**.

### 3.1 Adhesive Properties

Prolonging the residence time of NPs that is used as drug delivery systems at a target tissue results in a higher bioavailability of the drug and enables lower doses and increasing dosing intervals improving patient‐compliance. Therefore, thiolated NPs seem to be a valuable tool for drug delivery. They can adhere to various biological surfaces such as mucus, keratinous surfaces, extracellular matrix substructures or cell membranes. Adhesion of NPs is usually achieved via ionic interactions and secondary bonds including hydrogen bonding, dipole interactions and molecule–molecule interactions, whereas thiolated NPs can form covalent bonds. Either via thiol/disulfide exchange reactions or via a simple oxidation process, they can form disulfide bonds with disulfide and thiol substructures of biological surfaces. These properties were first discovered on mucosal surfaces.^[^ ^17^ ^]^ As most mucus glycoproteins display cysteine‐rich subdomains the mucus gel layer covering mucosal surfaces is predestined to covalently anchor thiolated NPs. Evidence for the formation of new disulfide bonds between thiolated polymers and mucus glycoproteines was first provided by Leitner et al.^[^ ^130^ ^]^ Since then numerous types of thiolated polymers have been generated and tested in clinical trials regarding their mucoadhesive properties.^[^ ^11^ ^]^ Eye drops containing a thiolated chitosan, for instance, significantly increased mean tear film thickness in patients with dry eye syndrome for even 24 h providing strong evidence for pronounced mucoadhesive properties.^[^ ^131^ ^]^ These and other clinical trials led to the development of first already marketed products such as Lacrimera eye drops.^[^ ^132^ ^]^ When thiolated polymers are formulated to NPs these polymers maintain their mucoadhesive properties.^[^ ^133^ ^]^ Dünnhaupt et al., for instance, compared the mucoadhesive properties of various polymeric NPs. Generally, thiolated polymeric NPs exhibited at least sixfold higher mucoadhesive properties than the corresponding unthiolated NPs. Furthermore, thiolated chitosan NPs had greater mucoadhesive properties than thiolated polyacrylate NPs.^[^ ^7^ ^]^ This observation might be explained by the cationic groups of chitosan that can ionically interact with sialic acid and sulfonic acid substructures of mucus glycoproteins, whereas polyacrylates cannot interact ionically with mucus glycoproteins. Trimethyl chitosan–cysteine conjugate (TMC–Cys) NPs containing insulin showed a 2.1–4.7‐fold increase in mucoadhesion compared to the same NPs but without conjugated cysteine.^[^ ^134^ ^]^

Apart from polymeric NPs even inorganic NPs exhibiting mucoinert properties can be made highly mucoadhesive by the attachment of thiol functional groups to their surface. In a series of studies, Khutoryanskiy and co‐workers demonstrated that thiolated organosilica NPs exhibit strong adhesion to ocular, gastrointestinal, and urinary bladder mucosa.^[^ ^88^, ^91^, ^135^ ^]^ However, when the thiol groups present on the surface of these NPs were substituted with short chains of polyethylene glycol or poly(2‐ethyl‐2‐polyoxazoline) these NPs lost their mucoadhesive properties but developed the ability for mucus‐penetration. Thiolated gold NPs (AuNPs) have also been demonstrated to show strong mucoadhesive properties. Ouellette et al. demonstrated that when two types of AuNPs were synthesized; one type with and one type without peripheral thiol groups that both showed varying degrees of mucoadhesion.^[^ ^136^ ^]^ Their study showed that both the AuNPs were able to bind the mucin, indicating that both the external and even the internal thiol groups of the AuNPs were able to form disulfide bonds with mucin.

**Figure 9:**
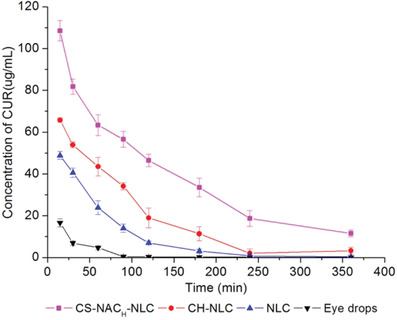
Ocular residence time of curcumin (CUR) being administered to rabbits in form of eye drops, nanostructured lipid carriers (NLC), NLC coated with chitosan (CH‐NLC), and NLC coated with thiolated chitosan (CS‐NAC‐NLC). Reproduced with permission.^[^ ^77^ ^]^ Copyright 2016, Springer.

The mucus gel layer covering mucosal membranes that are in contact with acidic media such as the gastric, upper intestinal, and vaginal mucosa, exhibits a pH gradient from acidic at the luminal site to pH 7.2 at the epithelial site. Thiol groups on the surface of all types of NPs become consequently more reactive the deeper they penetrate into such mucus layers, as more thiolate anions—representing the reactive species of thiols—are formed at high pH values. This pH‐gradient limits the binding of thiolated NPs to the loose outer mucus layer that is rapidly renewed by the mucus turnover process allowing NPs to penetrate into the deeper firmly bound mucus gel layer. On mucus gel layers without a pH‐gradient, however, less reactive thiols seem to be advantageous in order to avoid an extensive binding to outer mucus regions.^[^ ^137^ ^]^ Thiolated NPs can be additionally decorated with mucolytic enzymes in order to improve their mucus permeation behavior.^[^ ^16^ ^]^ Alternatively, thiolated polymers can be used as coating material for nanocarriers such as liposomes to generate high mucoadhesive properties.^[^ ^67^, ^74^, ^75^ ^]^ Thiolated polymers with hydrophilic and lipophilic substructures are directly used for the formation of polymeric micelles. The mucoadhesive properties of such micelles were also shown by numerous research groups.^[^ ^32^, ^36^, ^138^ ^]^ Dufresne et al. were likely the first providing evidence for the formation of disulfide bonds between thiol functionalized polymeric micelles and mucins.^[^ ^138^ ^]^ Moreover, thiolated surfactants as listed in Table 2 were shown to improve the mucoadhesive properties of lipid‐based NPs when they are anchored in oily phase of these formulations with their hydrophobic tail. For example, Shen et al. used a thiolated PEG stearate to improve the ocular residence time of nanostructured lipid carriers for cyclosporine delivery. Both precorneal residence time and drug concentration were dramatically increased compared with the same formulation without the thiolated surfactant.^[^ ^58^ ^]^ Due to a prolonged mucosal residence time of thiolated NPs also the mucosal residence time of the drug per se being released from these NPs can be prolonged. Ideally, the drug is continuously released from these formulations as long as the NPs adhere to the mucosa. The longer thiolated NPs adhere to the mucosa the less often the drug needs to be administered. Liu et al., for instance, investigated the ocular residence time of the model drug curcumin on the ocular surface of rabbits. As shown in **Figure** **9**, thiolated NPs providing a sustained drug release guaranteed the highest drug concentration on the target mucosa over the longest period of time.^[^ ^77^ ^]^

On mucosal membranes that exhibit a more rapid mucus turnover such as the gastrointestinal mucosa, however, it is challenging to achieve a prolonged residence time.

Apart from adhesion to mucus glycoproteins, adhesion of thiolated NPs was also shown to numerous other endogenous proteins. NPs with thiol groups on their surface are expected to remain bound to the surface of skin and hair as they are rich in keratin. Polyacrylic acid was modified with sulfhydryl moieties to improve its binding to the skin, resulting in a 15‐fold increase in adhesion compared to the unmodified polymer.^[^ ^139^ ^]^ In another study cysteine modified pluronic F127 micelles were shown to provide a prolonged dermal retention by formation of disulfide bonds with keratins.^[^ ^140^ ^]^ Similarly, thiolation of silicone oil improved adhesion to the skin in comparison to commonly used silicone oils.^[^ ^141^ ^]^ Targeted drug delivery to hair follicles is valuable for treating conditions such as alopecia and acne, and this shunt pathway may also allow drug delivery to deeper layers of the skin and systemic circulation, avoiding the intact stratum corneum. Reulen et al. generated thiolated liposomes by using a cysteine‐functionalized phospholipid as shown in Table 2 demonstrating the binding of collagen via the formation of disulfide bonds.^[^ ^62^ ^]^

### 3.2 Redox Triggered Functionalities

Redox‐triggered systems are systems that take advantage of reductive environments such as those routinely found in intracellular regions, in certain types of diseased tissues like cancer or within the colon. By making use of such reductive environments drugs can be released in a targeted manner, bioinert coatings can be detached from NPs at the target site favoring their cellular uptake and NPs can convert their zeta potential to positive in order to overcome the polycation dilemma.^[^ ^5^ ^]^

#### 3.2.1 Targeted and Controlled Drug Release

The possibility to target and to control drug release from thiolated NPs in a redox‐triggered manner opens up new opportunities for drug delivery. By a targeted drug release the amount of drug to be administered can be lowered and toxic side effects can be minimized.

A redox‐triggered drug release can be achieved by the entrapment of drugs in thiolated NPs that are crosslinked via disulfide bonds. Under reducing conditions these disulfide bonds are broken and the entrapped drug is released because of a dissociation of NPs or alterations in their assembly. In particular the interest in cytosolic delivery has prompted the development of redox‐triggered thiolated NPs. As the concentration of glutathione is much higher in the cytosol (≈2 × 10^−3^–10 × 10^−3^ m) than in the extracellular environment (1.5 × 10^−6^ m), the intracellular redox potential can trigger the release of drugs in the cytosol. The concept goes back in the 1980s when liposomes were additionally stabilized via a disulfide crosslinking that was reversible under reducing conditions.^[^ ^54^ ^]^ In the following the potential of such liposomes was utilized for a targeted release of plasmid DNA into the cytosol.^[^ ^142^ ^]^ The concept was then transferred to numerous other types of nanocarriers.

Alternatively, drugs exhibiting thiol substructures such as therapeutic peptides and certain anticancer drugs can be covalently attached to thiolated NPs via disulfide bonds. Under reducing conditions, these disulfide bonds are broken releasing the drug. The neuropeptide secretoneurin, for instance, was modified by the introduction of a cysteine residue to gain higher stability against enzymatic degradation and to bind the peptide via a disulfide bond to thiolated chitosan NPs. Under the hypoxic conditions of limb ischemia this therapeutic peptide was released at the target site by disulfide cleavage.^[^ ^143^ ^]^ The anticancer drug camptothecin was conjugated via a cleavable disulfide bond linker to palmitic acid and encapsulated into solid lipid nanoparticles. Under reducing conditions similar to those in the microenvironment of many tumors the drug was released from the thiolated NPs.^[^ ^56^ ^]^ In another study the anticancer drug 6‐mercaptopurine was covalently attached to branched poly((S‐(4‐vinyl) benzyl S′‐propyltrithiocarbonate)‐*co*‐(poly(ethylene glycol) methacrylate)) via a disulfide linkage. In aqueous media this conjugate self‐assembled into amphiphilic micelles that disassembled under reducing conditions releasing the drug.^[^ ^144^ ^]^

#### 3.2.2 Release of PEG‐Coatings

**Figure 10:**
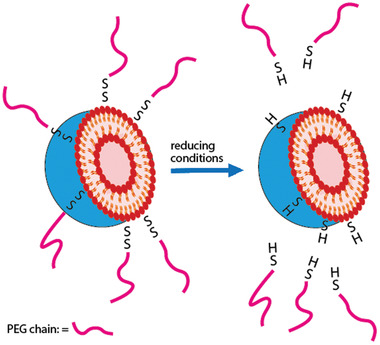
The cleavage mechanism of disulfide bonds‐linked PEGylated liposomes.

In the systemic circulation serum proteins like albumin, apolipoproteins, immunoglobulins, and complement components are adsorbed on the surface of NPs. This phenomenon—known as opsonization—favors the uptake of NPs by the mononuclear phagocytic system.^[^ ^145^ ^]^ The formation of this protein corona depends on numerous factors such as surface charge and lipophilicity.^[^ ^146^ ^]^ In the presence of a hydration layer on the surface of NPs, however, protein adsorption can be effectively lowered.^[^ ^145^ ^]^ In particular, PEG‐coatings are highly efficient antifouling materials that can be regarded as gold standard to provide stealth properties and to ensure subsequently prolonged blood circulation of NPs. When PEG‐coated NPs have reached their target site, however, the PEG‐coating hinders their cellular uptake, as these NPs are then also bioinert toward the cell membrane. In order to overcome this so‐called PEG dilemma, NPs with a PEG‐coating that is detached from NPs at the diseased tissue as illustrated in **Figure** **10** were developed making use of redox‐triggered cleavable disulfide linkages.^[^ ^147^ ^]^

The concept was first used to PEGylate therapeutic proteins in order to maintain their structure and biological activity unaltered after entering the cell^[^ ^148^ ^]^ and then transferred to NPs. In particular liposomes that were coated with PEGs being anchored on the surface via disulfide bonds showed potential.^[^ ^149^, ^150^, ^151^ ^]^ Under reducing conditions the PEG corona detached exposing the cationic surface of the liposomes facilitating internalization into diseased target cells.^[^ ^151^ ^]^ In the following even dual responsive systems depending on a redox‐ and pH‐triggered mechanism were designed.^[^ ^152^ ^]^ For gene delivery especially the disulfide linkage of PEG to cationic polymers showed potential for the design of nonviral delivery vectors.^[^ ^153^, ^154^ ^]^ In order to meet the special needs of gene transfection, Cai et al. designed mPEG‐SS‐poly‐l‐lysine/DNA complexes showing increased intracellular uptake under reducing conditions removing the outer PEG shell.^[^ ^153^ ^]^

Although cationic NPs can show a tremendously improved cellular uptake in vitro, their in vivo performance is rather disappointing. Because of their cationic character they are either immobilized on anionic surfaces on the way to their target or are getting coated by anionic components reaching their target without a cationic charge. A promising strategy to overcome this so‐called polycation dilemma is delivery systems that can convert their surface charge to positive once having reached their target cell. Apart from cationic surface decorations that can be cleaved by target specific enzymes, thiolytic‐cleavable coatings are employed for the design of charge converting systems. Redox‐triggered PEG releasing coatings as described above were shown to mask the cationic surface charge of NPs. The cationic charge of TAT‐decorated liposomes, for instance, was masked by long‐chain PEGs having been attached to the surface via a disulfide bond. Under reducing conditions at the target site these PEG chains were detached resulting in a threefold improved cellular uptake.^[^ ^155^ ^]^

### 3.3 Cellular Uptake Enhancing Properties

The cell uptake mechanism is mainly based on different reactions between thiol functions on the carrier surface with exofacial thiols of transmembrane proteins resulting in an increased absorptive endocytosis of API‐loaded thiolated carriers.^[^ ^9^ ^]^ Disulfide exchange reaction between a disulfide group at the carrier and a thiol group of the transmembrane proteins or the other way round can bind the carrier to the membrane protein. Moreover, a permeation enhancement as well as mucoadhesive properties of thiols displayed on the nanocarrier surface contributes to the cell uptake efficiency. The tight junction opening as previously described can increase the total cell surface area and therewith enable the access of the delivery system to the basolateral surface as well, whereas the mucoadhesive properties prolong the interaction time and therewith increase the chance of cell uptake over time.^[^ ^13^ ^]^

As cellular uptake is controlled by tightly regulated mechanisms, it is possible to exploit these natural mechanisms with rationally designed NPs. Thiol groups naturally present on the cell surface (exofacial thiols) can be used to enhance cellular association and internalization of thiolated NPs.^[^ ^9^ ^]^ Thiolated chitosan NPs containing the gene reporter for secreted alkaline phosphatase showed a fivefold increase in protein expression when compared to unmodified chitosan NPs.^[^ ^156^ ^]^ Martien et al. designed thiolated chitosan NPs as oral gene delivery system achieving also a fivefold improved transfection efficacy. This effect, however, was also related to the improved stability of the plasmid toward nucleases.^[^ ^157^ ^]^ Similar results were reported by Jiang et al. showing improved cellular uptake of an anticancer drug with thiolated chitosan NPs.^[^ ^158^ ^]^

Kichler et al. explored the possibility of exploiting the presence of thiol‐reactive phospholipid derivatives^[^ ^159^ ^]^ on lipospermine/DNA particles by complexing them with galactose ligands to make them selective for cells expressing the Gal/GalNAc receptor. Transfection efficiency was found to be increased by hundreds times due to the presence of thiol‐reactive functions (maleimide, bromoacetamide) leading to covalent binding with cell surface thiol groups and subsequent endocytosis of the complex.^[^ ^159^, ^160^ ^]^ Thiolation can be used not only to increase transfection efficiency, but also by improving cytotoxicity values. Indeed, Kang et al. synthesized a low molecular weight polycationic polyethyleneimine through thiolation processes, which made it 10–20 times less cytotoxic and able to complex plasmid DNA. Thiols/polyenes exploit polymeric degradation induced by membrane redox potential involving disulfide bonds, giving 1200–1500‐fold higher transfection efficiency than control.^[^ ^161^ ^]^ The extracellular redox potential can be controlled by several membrane proteins containing thiol groups (disulfide isomerases, thyroxidines), which also facilitate the process of cellular endocytosis of disulfide‐containing NPs.^[^ ^162^ ^]^ This process was investigated by Eksteen et al. through the incorporation of a thiol group into the structure of histidine‐rich peptides used for the preparation of nanocarriers directed to cancer cells.^[^ ^163^ ^]^ The disulfide linker used to bind carrier and drug allows binding to exofacial thiols, promoting endocytosis.^[^ ^113^ ^]^ The reaction is also facilitated by the presence of zinc at the neovascular level and extracellular redox potential that, through the action of the thiol, trigger the intracellular delivery of the peptide at the lysosomal membrane inducing cell death.^[^ ^163^ ^]^

### 3.4 Permeation Enhancing Properties

The permeation enhancing effect of thiolated polymers—designated thiomers—is based on epithelial tight junction (TJ) opening via interaction with thiol groups of membrane bound enzymes and proteins according to different mechanisms.^[^ ^164^, ^165^ ^]^ Clausen et al. described a glutathione mediated inhibition of protein tyrosine phosphatase by thiomers. The inhibition of this enzyme blocks the dephosphorylation of tyrosine subunits on occludin leading to a TJ opening.^[^ ^164^ ^]^ Recently, Zhang et al. supported an interaction of thiomers with membrane receptors like epidermal growth factor and insulin‐like growth factor, resulting in an activation of proto‐oncogene tyrosine‐protein kinase due to phosphorylation and further disruption of claudin‐4 leading to TJ opening. Thiomers have several advantages over most other permeation enhancers. For example, a more pronounced enhancing effect has been demonstrated for thiolated polycarbophil compared to the same concentration of sodium caprate—the representative oral permeation enhancer.^[^ ^166^ ^]^ Due to their high molecular mass, thiomers cannot be adsorbed and systemic effects can be avoided in contrast to smaller permeation enhancers. Moreover, it has been shown that the permeation‐enhancing effect induced by thiols is reversible.^[^ ^167^ ^]^ Compared with free thiols, S‐protected thiols show an even higher permeation‐enhancing effect.^[^ ^165^, ^168^, ^169^ ^]^ The permeation enhancing properties of thiomers were already utilized in numerous nanoparticulate formulations. Saremi et al., for instance, investigated carriers for docetaxel (DTX) with a thiolated chitosan shell and found an increased oral bioavailability of 68.9% compared to oral DTX with only 6.5% serving as positive control.^[^ ^170^ ^]^ A specific permeation across a Caco‐2‐cell monolayer could be achieved with DTX‐loaded NPs compared to DTX itself. In another study a pronounced effect of FD4 loaded thiolated NPs with a 3.0–5.3‐fold enhanced permeation in comparison to FD4 without the thiolated formulation was shown.^[^ ^22^ ^]^ More recently, Zhou et al. developed chitosan NPs coated with preactivated polymers. Their investigations showed improved paracellular transport of insulin across the intestinal epidermal cell layer, further improved mucus permeation behavior, and overall increased oral insulin bioavailability of up to 16.2%.^[^ ^171^ ^]^

### 3.5 Efflux Pump Inhibitory Properties

Limitations in bioavailability of anti‐inflammatory drugs, anticancer drugs, and antibiotics are often based on efflux pumps such as multidrug resistance proteins and P‐glycoprotein (P‐gp). Inhibition of this transport mechanism is an important strategy for drug delivery systems. Therefore, the use of thiolated polymers is advantageous as they show a reversible inhibition of efflux pumps via disulfide formation with cysteine‐substructures of endogenous proteins located in the channel of these pumps.^[^ ^172^, ^173^, ^174^ ^]^ Further, Föger et al. were able to demonstrate a higher inhibitory effect of thiolated polymers compared to other inhibitors such as the low molecular mass inhibitors GSH and 6‐mercaptopurine in rats.^[^ ^175^ ^]^ Grabovac et al. demonstrated that the extent of inhibition is dependent on the amount of thiols attached to the polymer as well as its molecular mass.^[^ ^172^ ^]^ Moreover, S‐protection of the thiomers via pyridyl substructures leads to higher thiol reactivity and thus more pronounced P‐gp inhibition.^[^ ^176^ ^]^ For example, Netsomboon et al. found a 2.36‐fold enhanced permeation of rhodamine 123 in the presence of preactivated poly(acrylic acid)‐cysteine‐2‐mercaptonicotinic acid compared to rhodamine 123 alone. In another study, the author was able to additionally provide evidence for the reversibility of the P‐gp inhibition by obtaining an apparent permeation coefficient (Papp) to rhodamine 123 alone after removal of the thiomer.^[^ ^177^, ^178^ ^]^ This efflux pump inhibition properties can also be utilized for nanocarrier systems. Chen et al. prepared self‐assembled NPs, consisting of arginine‐modified chitosan and thiolated fucoidan and demonstrated an increased permeability of hydrophilic macromolecules and hydrophobic compounds through a regulated tight junction opening as well as P‐gp inhibition.^[^ ^179^ ^]^ Huo et al. developed micelles loaded with paclitaxel containing thiolated chitosan and showed 3.8‐fold improved oral bioavailability of the API with this formulation compared to the marketed product Taxol.^[^ ^31^ ^]^

## 4 Applications of Thiolated Nanoparticles

### 4.1 Drug Delivery

**Figure 11:**
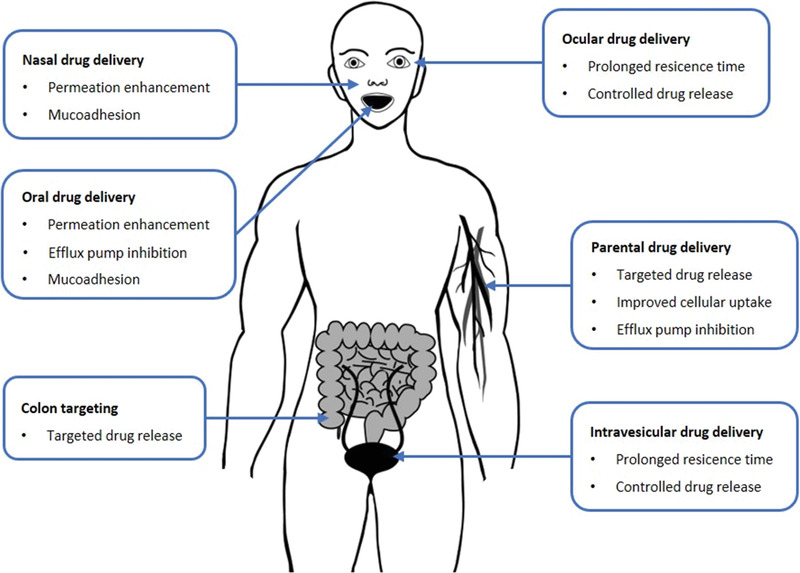
Applications of thiolated NPs for mucosal and parenteral drug delivery.

The properties of thiolated NPs such as adhesive properties, redox‐triggered functions, cellular uptake enhancing properties, permeation enhancing properties, and efflux pump inhibition make them highly potent delivery systems for numerous drug categories and routes of administration. As these key properties have already been described in detail above, we will focus in this section primarily on combined effect of different properties for various routes of administration and the overall in vivo performance of these delivery systems. An overview about the various applications of thiolated NPs as drug delivery systems is provided in **Figure** **11**.

**Figure 12:**
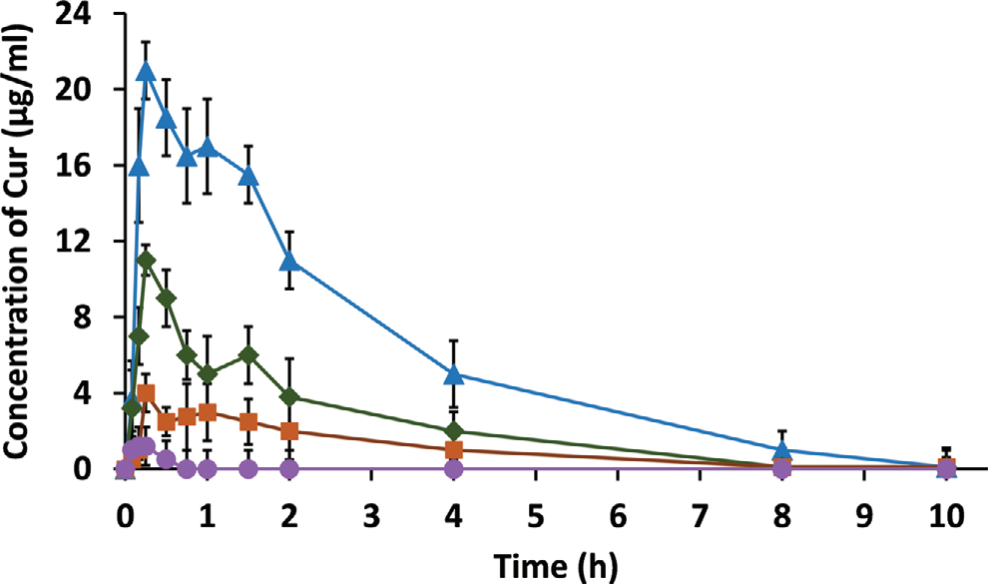
Oral bioavailability of curcumin (Cur) in solution (●), from unmodified NPs (■) and from NPs with low and high content of N‐acetyl‐l‐cysteine‐polyethylene glycol (low: ♦; high: ▲). Reproduced with permission.^[^ ^60^ ^]^ Copyright 2017, Taylor & Francis.

In case of systemic drug delivery via mucosal membranes in particular adhesive properties providing a prolonged residence time at the absorption membrane as well as permeation enhancing and efflux pump inhibiting properties are beneficial. Tian et al., for instance, synthesized N‐acetyl‐l‐cysteine‐polyethylene glycol (NAPG) conjugate providing mucoadhesive and permeation enhancing properties. Curcumin loaded NPs showed a 499‐ and 117‐fold increased drug absorption and oral bioavailability as illustrated in **Figure** **12** when having been functionalized with NAPG.^[^ ^60^ ^]^

**Figure 13:**
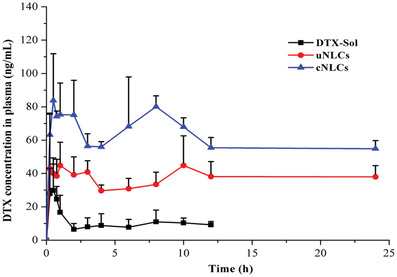
Plasma concentration profiles of docetaxel (DTX) after oral administration of the drug in solution (DTX‐Sol), unmodified nanostructured lipid carriers (NLCs), and cysteine‐modified NLCs in SD rats (*n* = 6). Reproduced with permission.^[^ ^59^ ^]^ Copyright 2015, American Chemical Society.

In another study cysteine was anchored on the surface of nanostructured lipid carriers containing DTX. Thiolation of these nanocarriers allowed a specific interaction with mucins and facilitated intestinal drug absorption. In vivo studies showed an improved intestinal absorption and a 12‐fold high oral bioavailability due to the use of this formulation. Results of this study are illustrated in **Figure** **13**.^[^ ^59^ ^]^

To what extent mucoadhesive, permeation enhancing and efflux pump inhibitory properties contribute to this improved bioavailability could not be determined, although it appeared that thiolated NLCs increase the interaction with the intestinal tract by forming disulfide bonds. Nonetheless, both studies provide strong evidence for the potential of thiolated NPs for oral drug delivery. In a similar way, the systemic uptake of nasally administered drugs can be essentially improved by the use of thiolated NPs. Brar and Kaur, for instance, prepared NPs with a thiolated polymer that provided an increased residence time of the esculin, a drug useful to treat Parkinson's disease, on the nasal mucosa (>4 h). Systemic drug uptake was thereby significantly improved.^[^ ^180^ ^]^ The potential of thiolated NPs for nasal drug delivery was also demonstrated in various other in vivo studies.^[^ ^181^, ^182^, ^183^ ^]^ Regarding ocular drug delivery in particular a prolonged mucosal residence time of the drug delivery system is advantageous. As illustrated in Figure 9 an ocular residence time even above 6 h can be achieved with thiolated NPs.^[^ ^77^ ^]^ Similar results were shown by Xu et al., although their thiolated NPs exhibited a comparatively shorter ocular residence time of around 3 h.^[^ ^184^ ^]^ On other mucosal membranes such as the intravesical mucosa the prolonged residence time of thiolated NPs seems to be most important as well.^[^ ^30^ ^]^

In case of local drug delivery to mucosal membranes in particular their redox‐triggered drug releasing properties are of relevance. With a redox potential of −67 ± 90 mV in the small intestine and −415 ± 72 mV in the right colon, reductive cleavage of disulfide bonds is more probable in the colon, as a disulfide bond shows a standard reduction potential of −250 mV.^[^ ^34^, ^185^ ^]^ Chang et al. developed thiolated alginate‐based nanoparticles with improved colonic targeting potential, demonstrated by a marked increase in drug release in a colonic pH mimic and reducing fluid.^[^ ^34^ ^]^ Providing a highly effective tool in the targeted treatment of colon cancer and inflammatory bowel disease, other research groups have followed and established thiolate alginate‐based nanocarriers for budesonide, docetaxel, and doxorubicin^[^ ^186^, ^187^ ^]^ In addition, paclitaxel (PTX)‐loaded NPs based on chitosan‐Eudragit S‐100 with disulfide bond (CSE NP) were developed by Sood et al.^[^ ^188^ ^]^ After 48 h of treatment of HCT116 colon cancer cells, they observed a uniform distribution of PTX within the cells and a significant accumulation in the G2/M phase after 24 h, indicating the arrest of cell division during the mitotic phase. In subsequent in vivo biodistribution studies in male Balb/C mice, a retention of NPs in the colonic region up to 24 h after oral administration provided strong evidence for colon‐specific targeting.

**Figure 14:**
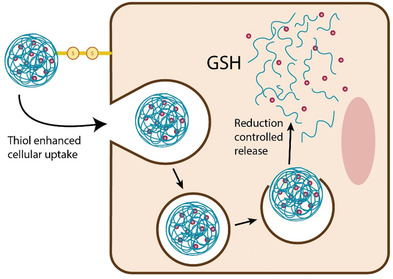
Schematic illustration of cellular uptake enhancing and release controlling mechanism of thiolated NPs.

In case of parenterally administered thiolated NPs especially their drug targeting and cellular uptake enhancing properties are beneficial. For parenteral delivery of DNA‐ and RNA‐based drugs sufficient endocytosis of NPs containing these drugs and their escape from the endosomal–lysosomal system are essential for their internalization and expression. Thiolated NPs can enhance both processes. On the one hand, thiol groups increase the cellular uptake of NPs and on the other hand a redox‐triggered drug release in the cytoplasm as illustrated in **Figure** **14** can facilitate escape from the endosomal–lysosomal system. Under the aid of thiolated NPs the transfection efficacy can be even 1000‐fold improved.^[^ ^161^ ^]^ Kakizawa et al. developed micelles based on a PEG‐SS‐antisense oligodeoxynucleotide conjugate for cytoplasmic delivery.^[^ ^189^ ^]^ Furthermore, they could achieve a 100‐fold higher siRNA transfection efficacy with disulfide crosslinked micelles containing thiolated PEG–poly(l‐lysine) in comparison to a control formulation.^[^ ^190^ ^]^ Since DNA‐ and RNA‐based drugs exhibit a polyanionic character they can be simply coacervated with thiolated cationic polymers and stabilized via disulfide bond formation as described in detail above. In this context, a wide range of thiolated cationic polymers such as poly(amido amines), poly(amino esters), oligomerized amines, and chitosan among others were formulated to thiolated NPs.^[^ ^11^ ^]^ Martien et al. were likely the first who designed thiolated chitosan NPs for oral gene delivery following this concept.^[^ ^157^ ^]^ Oliveira et al. used also a thiolated chitosan in order to improve the transfection efficiency of a DNA‐based drug showing the release of this cargo in the cytosol of retinal pigment epithelium cells.^[^ ^191^ ^]^ He et al. developed and characterized mannose‐modified trimethyl chitosan–cysteine conjugate NPs for oral delivery of TNF‐a siRNA. They showed a successful inhibited TNF‐a production in macrophages protecting mice with acute hepatic injury from inflammation‐induced liver damage and lethality.^[^ ^192^ ^]^ By using nanoparticles consisting of poly(amido ethylenimine) with multiple disulfide bonds, Christensen et al. achieved a 20‐fold higher transfection efficiency of plasmid DNA than with polyethylenimine control.^[^ ^193^ ^]^

Apart from DNA‐ and RNA‐based drugs, a targeted intracellular delivery is also for anticancer drugs of high relevance as in this way efflux pumps being responsible for drug resistance can be overcome. In contrast to normal cells cancer cells show up to 4‐fold higher glutathione concentrations.^[^ ^194^, ^195^ ^]^ Talaei et al., for instance, incorporated doxorubicin and an antisense oligonucleotide in thiolated chitosan NPs that were crosslinked via disulfide bond formation. In a simulated release/reduction environment from the cytosol, such as the intracellular environment, NPs dissociated, releasing ≈50% of both active substances within 7 h.^[^ ^20^ ^]^ Other research groups developed thiol‐responsive micelles based on nonionic gemini surfactants consisting of hydrophilic blocks of PEG and hydrophobic blocks of polylactide with a rigid or flexible disulfide spacer consisting of cystine with a cystine disulfide spacer in order to improve the intracellular delivery of doxorubicin.^[^ ^196^, ^197^, ^198^, ^199^ ^]^ In the presence of glutathione as a reducing agent, gemini micelles gradually destabilize into monomeric micelles through the cleavage of cystine binding. This destabilization of gemini micelles changed their size distribution, with the appearance of small aggregates, and led to increased release of encapsulated doxorubicin.^[^ ^200^, ^201^, ^202^ ^]^ Another research group demonstrated in in vitro studies a significant cytotoxic effect for cancer cells but a safe profile for healthy human liver cells by applying thiolate sodium alginate‐based nanocarriers for doxorubicin.^[^ ^203^ ^]^ In addition, they were able to demonstrate high payload release selectivity to tumor cells and good stability in healthy cell lines, avoiding untargeted drug leakage.

In many tumors reducing conditions cannot be found just in intracellular regions but also in the intercellular space. The payload of thiolated NPs might therefore be already released before these carriers have reached the cytosol of target cells. This reducing extracellular environment, however, can be used for a targeted release to solid tumors. Micelles containing doxorubicin were designed with a heparin precursor that was conjugated to vitamin E via a disulfide linkage. These drug‐loaded micelles had an average particle size of 90–120 nm and showed high stability in serum. Under reducing conditions as found in the microenvironment of solid tumors, however, they depolymerized releasing their payload.^[^ ^204^ ^]^ Qiu et al. studied polypeptide nanoparticles with doxorubicin for tumor targeting. They found drug accumulation at tumor sites with significantly reduced side effects, and improved quality of life of tumor‐bearing mice.^[^ ^35^ ^]^

### 4.2 Biosensoric and Diagnostic Applications

Especially because of their small size and optoelectronic properties NPs such as noble metal NPs,^[^ ^205^ ^]^ magnetic NPs^[^ ^206^ ^]^ and QDs^[^ ^207^ ^]^ are useful tools for biosensoric and diagnostic applications. They can provide rapid and highly site‐specific contrast enhancement and signal amplification. Some of them are routinely used in clinical practice for computer tomography, magnetic resonance imaging, photoacoustic imaging, optical imaging, single photon emission computer tomography, and positron emission tomography. Without specific surface modifications, however, the properties of such NPs are not fit for the intended application. Poor solubility in aqueous media, the tendency to aggregate or insufficient storage stability is just a few examples for shortcomings. In vivo, numerous further shortcomings such as too short circulation times, extensive uptake by the reticuloendothelial system, no or unspecific retention in the extravascular compartment contributing to high background signals limit their potential. Furthermore, as they should possess high selectivity to their target in order to outperform low‐molecular‐weight contrast agents, for which the unbound fraction is rapidly eliminated via renal clearance.^[^ ^208^ ^]^ In order to achieve appropriate signal‐to‐noise levels, pharmacokinetic properties, biodistribution, and tissue penetration the surface properties of these NPs have to be adjusted.^[^ ^209^ ^]^ Over the last decades, thiolation turned out as useful tool to decorate the surface of NPs in a comparatively simple and efficient manner. Thiolated ligands can on the one hand be efficiently anchored on the surface of noble metal NPs via the formation of Me—S bonds or on the other hand to thiol bearing NPs via disulfide bond formation.

Utilizing the strong Pd—S, Au—S, and Ag—S binding, palladium, gold, and silver NPs were decorated with various thiol ligands.^[^ ^205^ ^]^ To avoid aggregation, improve solubility in aqueous media and to provide bio‐inert properties in order to prolong circulation half‐life, to reduce uptake into the reticuloendothelial system and to improve their diffusivity in target tissues in particular PEG coatings with thiol‐terminated polyethylene glycols (PEG‐SH) of different chain length are well established. Main advantages of thiolated PEGs are the easy synthesis, ease of handling, and the versatile applications. Alalaiwe et al., for instance, coated gold NPs with thiol‐terminated 1, 2, and 5 kDa PEG showing that the elimination half‐life after intravenous administrations decreases from 17.4 h, to 13.2 h and 7.3 h, respectively, with increasing PEG chain length.^[^ ^210^ ^]^ As gold can be easily integrated into SPIO NPs via electron beam irradiation of a solution of Au and SPIO, the same approach can also be used for a PEG‐coating of SPIOs. Kojima et al., for instance, conjugated a thiol‐terminated PEG to the surface of Au/SPIO NPs via an Au—S bond without the need for any chemical reactions providing evidence that PEG‐modified SPIO NPs can be prepared on an industrial scale with low cost.^[^ ^211^ ^]^ Furthermore, targeting ligands such as antibodies can be attached to these NPs by making use of the strong Me—S bond. Free sulfhydryl groups of antibodies can be used to anchor these targeting ligands to noble metal NPs. By using a dip coating/atomic force microscopy technique the adhesion forces of an antibody to gold NPs were determined. Results showed a three times higher adhesion force between gold and a breast specific antibody, when it was anchored via a thiol substructure.^[^ ^212^ ^]^ Optionally antibody‐PEG spacer conjugates are utilized that a bound to the surface of NPs via a sulfhydryl group being located at the distal end of the PEG spacer.^[^ ^213^ ^]^ Other targeting ligands such as aptamers can also be functionalized with thiol groups and attached to noble metal NPs.^[^ ^214^ ^]^ Apart from thiolated ligands even thiolated graphene QDs can be attached to the surface of noble metal NPs. Valipour and Roushani, for instance, prepared thiolated graphene QDs that were subsequently attached to silver NPs.^[^ ^215^ ^]^ QDs are not only composed of cadmium selenide but of many other semiconducting materials derived from the II and VI elemental groups and III and V elemental groups of the periodic table. They can be coated with Zn‐containing shells exhibiting a much higher affinity to thiol groups than the mere core material. Thiol bearing ligands as described above can therefore also be attached to the surface of QDs. One of the easiest ways to obtain water‐soluble QDs is the attachment of thiolated polyethylene glycol.^[^ ^216^ ^]^ In another study the sulfhydryl compound dihydrolipoic acid was used as linker to bind antibodies to the surface of CdSe–ZnS core–shell NPs.^[^ ^217^ ^]^

**Figure 15:**
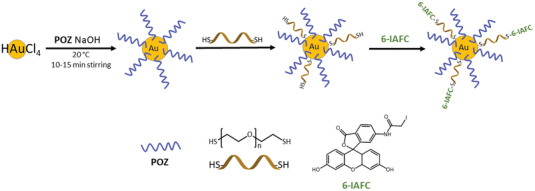
Scheme of synthesis of with 6‐(iodoacetamido)fluorescein (6‐IAFC) fluorescently‐labeled gold NPs decorated with poly(2‐ethyl‐2‐oxazoline) (POZ).^[^ ^221^ ^]^

In addition, thiol groups are attached to NPs that are used for biosensing and diagnostic approaches in order to facilitate the binding of different thiolated ligands such as antibodies or fluorescent dyes via disulfide bond formation. The introduction of thiol substructures to the surface of SPIOs with meso‐2,3‐dimercaptosuccinic acid, for instance, is a common method. Because of the chelating properties of the succinic acid substructure, the compound can be firmly attached to these iron oxide NPs presenting the free thiol groups for conjugation with thiolated ligands on the surface.^[^ ^218^, ^219^, ^220^ ^]^ Dithiols serve for the same purpose. Polyethylene glycol dithiol, for instance, can be anchored with one thiol substructure to noble metal NPs while presenting the other thiol substructure on the surface for disulfide bond formation with thiolated ligands. Recently, Nurgaziyeva et al.^[^ ^221^ ^]^ reported the synthesis of poly(2‐ethyl‐2‐oxazoline) protected gold NPs, which were subsequently thiolated by reaction with polyethylene glycol dithiol as illustrated in **Figure** **15**. The thiol groups on the surface of these NPs were then used to react with 6‐(iodoacetamido)fluorescein (6‐IAFC) to make fluorescently labeled gold NPs.

Furthermore, NPs can be coated with thiolated polymers that are optionally crosslinked via disulfide bond formation in order to increase the stability of such coatings. Vetter at el., for instance, coated SPIOs with thiolated polyacrylic acid in order to improve the uptake of this NPs by stem cells due to thiol‐mediated endocytosis for MRI stem cell imaging.^[^ ^222^ ^]^

## 5 Future Perspectives

As thiolated NPs are mimicking in many ways proteins representing the workhorses of our body, they are an ever‐expanding area with attractive future perspectives. Apart from certain cysteine‐rich proteins such as mucins and keratins, adhesion of thiolated NPs to numerous further cysteine‐rich substructures that are of therapeutic or diagnostic relevance will be discovered. Integrins, for example, being part of the likely most important cell adhesion mechanism bind and mechanically fix cells to extracellular matrix. As these proteins bear numerous cysteine‐rich domains with in total 56 cysteine residues on their extracellular *β*‐subunit,^[^ ^223^ ^]^ they might be an interesting target for the adhesion of thiolated NPs. Furthermore, thiol‐mediated bioadhesion of NPs might be utilized for even more targeted drug delivery. Since various tumor cells express a much higher amount of thiols on their surface,^[^ ^224^ ^]^ they might be a promising target for a selective and specific adhesion of thiolated NPs. Also in case of redox‐triggered properties there are numerous further options for sound applications. Hydrogen sulfide playing as cellular signaling agent a key role in numerous pathological processes is believed to be involved in physiological processes such as inflammation, angiogenesis, and apoptosis.^[^ ^225^ ^]^ It is therefore of high therapeutic relevance. As so far established hydrogen sulfide releasing systems cannot guarantee a controlled release of this therapeutic agent, more efficient delivery systems are needed. Thiolated NPs might provide the demanded controlled and sufficiently sustained release of hydrogen sulfide. Acyl‐protected per‐thiomers were already shown to release hydrogen disulfide in a sustained and controlled manner under reducing conditions in the presence of l‐cysteine.^[^ ^226^, ^227^ ^]^ Taking also the high bioadhesive properties of thiolated NPs into consideration not only a sustained but even a targeted release of hydrogen sulfide at diseased tissue seems feasible. Regarding biological processes that are triggered by thiolated NPs such as an enhanced cellular uptake^[^ ^156^ ^]^ we have likely taken just the very first steps. Taking the huge number of thiol‐triggered processes into consideration there are numerous further options in front of us. Taken all, thiolated nanoparticles will certainly further shape the landscape in biomedicine.

## Conflict of Interest

The authors declare no conflict of interest.
